# Associations of benzodiazepine use with cognitive ability and age-related cognitive decline

**DOI:** 10.1017/S0033291724002046

**Published:** 2024-10

**Authors:** Merete Osler, Maarten Pieter Rozing, Ida Kim Wium-Andersen, Martin Balslev Jørgensen, Erik Lykke Mortensen, Gunhild Tidemann Okholm

**Affiliations:** 1 Center for Clinical Research and Prevention, Bispebjerg and Frederiksberg Hospitals, Frederiksberg, Copenhagen, Denmark; 2 Department of Public Health, Section of Epidemiology, University of Copenhagen, Copenhagen, Denmark; 3 Department of Public Health, The Research Unit for General Practice and Section of General Practice, University of Copenhagen, Copenhagen, Denmark; 4 Psychiatric Center Copenhagen, Copenhagen, Denmark; 5 Institute of Clinical Medicine, University of Copenhagen, Denmark; 6 Department of Public Health, Section of Environmental Health, University of Copenhagen, Copenhagen, Denmark; 7 National Institute of Public Health, University of Southern Denmark, Copenhagen, Denmark

**Keywords:** benzodiazepines, cognitive ability, cognitive decline, cohort study

## Abstract

**Background:**

It remains uncertain whether long-term use of benzodiazepines is associated with age-related cognitive decline, and if cognitive ability in early life is the driver of any association. This study examines the association of cognitive ability in young adulthood with later use of benzodiazepines and explores whether the use of benzodiazepines during adult life is associated with cognitive decline in late midlife.

**Methods:**

The study samples include cognitive tests on the Børge Priens Prøve (BPP) from the conscription board examination (age 19 years) from 335 513 men born 1949–1961 and data from re-examinations of 5183 men 44 years later. Cognitive decline was defined as the difference between scores at the conscription board and the re-examination. Information on purchases of benzodiazepines was obtained from the Danish National Prescription Registry, 1995–2022. Associations were analysed using Cox proportional hazards and linear regression.

**Results:**

In total, 120 911 (36%) men purchased benzodiazepines during a follow-up of 20 years. Lower cognitive scores in young adulthood were associated with a higher risk of initiating benzodiazepines (hazard ratio [95% CI] = 0.71[0.68–0.75]). Men with the highest cumulative use of benzodiazepines had larger cognitive decline (*β*-coefficient [95% CI] = −1.66 [−2.09 to −1.23] BPP scores) compared with never users. Current benzodiazepine users showed a larger cognitive decline than never users (*β*-coefficient [95% CI] = −2.42[−3.18 to −1.66] BPP scores) and this partially explained the above association. These estimates for cognitive decline were relatively small and may lack clinical relevance.

**Conclusion:**

Low cognitive ability increases the risk of benzodiazepine use in adulthood and cognitive decline is more pronounced in those with the highest benzodiazepine use compared with never-use, but the difference lacks clinical significance.

## Background

Benzodiazepines and benzodiazepine-related drugs (Z-drugs) – hereafter referred to as benzodiazepines – are among the most prescribed medications globally and are used primarily for the management of anxiety and insomnia (Rosenqvist, Osler, Wium-Andersen, & Wium-Andersen, 2022). Several single-dose studies have shown that benzodiazepines provoke both amnestic and non-amnestic cognitive impairment, especially in older populations (Tannenbaum, Paquette, Hilmer, Holroyd-Leduc, & Carnahan, 2012) but it remains uncertain whether long-term use is associated with age-related cognitive decline. Some recent studies have suggested that long-term use does not increase the risk of cognitive decline, but results are conflicting (Gray et al., 2016; Mura et al., 2013; Picton, Marino, & Nealy, 2018; Verdoux, Lagnaoui, & Begaud, 2005). Further, the current evidence relies on studies conducted in populations aged 65 years or above with less than 8-year intervals between cognitive assessments. Cognitive ability in old age is affected by several factors operating across the life course. Moreover, low cognitive ability in childhood or young adulthood has been associated with several behavioural and medical conditions that are thought to accelerate cognitive decline, such as substance use disorder, accidents, unhealthy behaviors, and mental disorders (Batty, Deary, & Macintyre, 2007; Deary, Hill, & Gale, 2021; Osler, Andersen, Laursen, & Lawlor, 2007a; Osler, Lawlor, & Nordentoft, 2007b; Wraw, Der, Gale, & Deary, 2018; Zammit et al., 2004). As such, cognitive ability in early life might, to some extent, be the driver of the association between benzodiazepines and cognitive scores in late middle-age. This highlights the need for baseline measures of cognitive ability to be assessed sufficiently early in life when investigating predictors of cognitive decline. Unfortunately, there is a scarcity of aging cohorts that include early life measures of cognitive ability, and the ones that do, are often limited in size. For instance, the influential Lothian birth cohort contains only 550 and 1090 participants in the first waves of cognitive follow-up (Deary et al., 2009).

In Denmark, cognitive testing has been part of the conscription board examinations since 1957, and the Danish Conscription Database (DCD) has been established to explore early-life cognitive ability as a predictor of later-life health outcomes (Christensen et al., 2015). Further, a sample of men having cognitive test scores from the conscription board examinations has been invited at age 55–70 years to re-take the military intelligence test to assess cognitive decline from young adulthood to late midlife in the Danish Aging and Cognition (DanACo) cohort (Grønkjær et al., 2024).

In the present study, we use these unique data to investigate the hypotheses that: (1) lower cognitive ability in young adulthood is associated with a higher incidence of benzodiazepine use in adult men, and (2) cumulative use of benzodiazepines during adult life is associated with increased age-related cognitive decline in late midlife.

## Methods

### Population

Our study was based on data from the DCD and DanACo which have been described in detail elsewhere (Christensen et al., 2015; Grønkjær et al., 2024). In brief, the DCD holds information from the mandatory Danish conscription board examinations from 1957 through 1984 on cognitive test scores and educational level (at a median age of 19 years). From this database, we obtained information on all men born between 1949 and 1961, corresponding to the birth years of the invited participants of the DanACo cohort. Participants in the DanACo cohort were mainly sampled from the DCD and another database with conscription board information established by Urfer-Parnas et al. (Urfer-Parnas, Lykke Mortensen, Saebye, & Parnas, 2010). During the period 2015–2022, 37 444 men were invited to participate in a follow-up assessment (at a mean age of 64.4 years) consisting of a re-administration of the same intelligence test and a comprehensive questionnaire covering demographic, lifestyle, and health-related factors. A total of 5340 men participated, resulting in an overall participation rate of 14.3%. Online Supplementary Table 1 shows the distribution of conscription data among participants and non-participants in DanACo. Unfortunately, follow-up data from 157 participants were lost due to technical problems with the military's computer system, leaving a total of 5183 participants. However, the data was lost randomly in the subsample of 157 men and did not vary from the remaining men in DanACo.

### Data

Using the unique personal identification number assigned to all residents of Denmark, the cohorts were linked to data from the Danish National Prescription Registry and the Danish Psychiatric Central Research Registry for prospectively collected data on medication use and psychiatric hospital contacts.

Information on the use of benzodiazepines was obtained from the Danish National Prescription Registry, which contains information on all prescribed drugs dispensed from Danish pharmacies since 1995, the date of prescription redemption, and the Anatomical Therapeutic Chemical (ATC) code (Pottegard et al., 2017). Medication dispensed in inpatient settings and emergency departments is not recorded. We used the following ATC codes to define benzodiazepines: N05BA; N05CD; N03AE01, N03AF01, and Z-drugs: N05CF. Benzodiazepines and Z-drugs were merged as they have pharmacological similarities and give similar side effects (Brandt & Leong, 2017). For the first hypothesis, information was collected from January 1, 1995, hereafter termed ‘index date’, until December 31, 2022, and we used first-time use as the outcome. To create our exposure measures for the analyses on cognitive decline (study hypothesis 2) we extracted information on the defined daily dose (DDD) indicating the cumulative dose during the period between the index date until the follow-up assessment and calculated the diazepam milligram equivalents (DMEs) using the scale reported in online Supplementary Table 2. DME standardization allowed us to compare benzodiazepines regardless of their relative potency. We categorized cumulative use as no use, 1–30 DME, 31–120 DME, and >120 DME based on the distribution and clinically meaningful cutoff points (Gray et al., 2016). Benzodiazepines were also classified into short-acting Z-drugs mainly used for insomnia and long/medium-acting benzodiazepines most often used for anxiety. The indications for prescribing benzodiazepines and their immediate adverse effects include impaired concentration and somnolence (Sanabria, Cuenca, Esteso, & Maldonado, 2021). This might affect cognitive testing among those who were under treatment at the follow-up examination, and we, therefore, created a measure of current use defined as filling a prescription for a benzodiazepine within 180 days before the re-examination.

**Cognitive ability** was assessed using the intelligence test Børge Priens Prøve (BPP), which has been used at the mandatory conscription board examinations since 1957 (Christensen et al., 2015; Teasdale, Hartmann, Pedersen, & Bertelsen, 2011). The BPP is a group-administered and timed intelligence test consisting of four subtests, including letter matrices (19 items), verbal analogies (24 items), number series (17 items), and geometric figures (18 items). The items have remained unchanged since its implementation in 1957, but the format was changed from paper-and-pencil to a computerized format in 2010. The test is highly correlated (*r* = 0.82) with the full-scale score of the widely used Wechsler Adult Intelligence Scale (WAIS) (Mortensen, Reinisch, & Teasdale, 1989; Teasdale et al., 2011). The total BPP score corresponding to the number of correct answers on the four subtests with a 0–78 score range was available from the conscription board and re-examinations. *Cognitive decline* was defined as the difference in BPP test score between the two cognitive ability assessments conducted in early adulthood and late midlife, respectively. The two test scores were highly correlated (Pearson *r* = 0.79) whereas the correlation between baseline test score and the difference was *r* = −0.36. Moreover, we used the commonly used half a standard deviation (s.d.) of the conscription test score (½s.d. = 4.5 BPP score points) as cutoff point for a relevant difference in test scores between conscription and the re-examination (Revicki, Hays, Cella, & Sloan, 2008; Norman, Sloan, & Wyrwich, 2003).

Information obtained from registers and questionnaires was included as potential confounders assumed to be associated with exposure and outcomes. Concerning the first study hypothesis, the analyses were conducted with adjustment for self-reported educational level at conscription and register-based information on any psychiatric hospital contact (Munk-Jorgensen & Mortensen, 1997) before the date of conscription and were stratified on birth year. The second hypothesis adjustment was guided by the Directed Acyclic Graph in online Supplementary Figure 1 and included young adult (baseline) test score, re-test interval (years), age at follow-up, self-reported information on years of education, alcohol use, and smoking from the follow-up questionnaire and register-based information on any psychiatric hospital contact prior to the index date.

### Statistics

First, the association between young adult cognitive test scores and ever use of benzodiazepines in adult life was analyzed using Cox proportional hazard models in the cohort including all conscripts born 1949–1961 who were alive at the index date (January 1, 1995). These men were followed from the index date until the first prescription of benzodiazepines, death, emigration, or end of follow-up (December 31, 2022), whichever came first. To explore whether participants and non-participants in DanACo were representative of the total population, we repeated the analysis stratified on participation status. This analysis was restricted to men who were alive at the time of invitation to the DanACo cohort (September 1, 2015). Second, the association between cumulative benzodiazepine use and age-related cognitive decline from early adulthood to late midlife was analyzed using linear regression with adjustment for covariates (baseline test score, re-test interval, age at follow-up, years of education, alcohol use, smoking, and any prior psychiatric hospital contact). Potential interactions with baseline BPP test scores or psychiatric hospital contact were first explored in stratified analysis and, if applicable, tested by including interaction terms and using likelihood ratio. This showed no statistically significant interaction with baseline BPP scores or having a psychiatric hospital contact prior to the index date. We also explored associations between type of benzodiazepine use and current use, respectively, with age-related cognitive decline.

## Results

### Young adult IQ and use of benzodiazepines in middle age

Of the 335 513 men from the DCD who were alive in 1995 (age 34–45 years), 36% (*n* = 120 911) had purchased benzodiazepines during a mean follow-up interval of 20 years. Cognitive ability in young adulthood showed an inverse association with the purchase of any benzodiazepine. Thus, the risk of having purchased these drugs decreased with each s.d. increase in BPP test score at conscription after adjustment for the educational level at conscription and psychiatric hospital contacts prior to the index date (hazard ratio = 0.85 [95% CI 0.84–0.86]) (Table 1). The 5183 men who had participated in DanACo had higher mean BPP scores in young adulthood (46.6 [s.d. 9.5]) than the men who did not participate (39.8 [s.d. 11.4]). However, similar inverse associations between cognitive ability in young adulthood and later use of benzodiazepines were found when the analysis was stratified on participation status (online Supplementary Table 1).

**Table 1.** Associations of cognitive ability in young adulthood and use of benzodiazepines in middle age among Danish men born 1949 through 1961

	Total *n*	Benzodiazepine use *n* (% of total)	Crude hazard ratio (95% CI)	Adjusted* hazard ratio (95% CI)	Adjusted* hazard ratio (95% CI)
Total	335 513	120 911 (36.0)	–	–	–
Birth year, mean (s.d.)	1954 (3.0)	1954 (3.0)	–	–	–
Conscription board examination					
*BPP-score*, mean (s.d.)	39.1 (11.4)	37.8 (11.5)	0.86(0.86–0.87)**		0.85 (0.84–0.86)**
Low	121 245	49 012 (40.4)	Ref	Ref	
Medium	109 485	38 569 (35.2)	0.78 (0.77–0.80)	0.84(0.81–0.88)	
High	104 783	33 330 (31.8)	0.68 (0.87–0.69)	0.71 (0.68–0.75)	
*Educational level*					
Low	76 685	31 322 (40.8)	Ref	Ref	Ref
Medium	188 640	66 153 (35.1)	0.78 (0.77–0.79)	0.83(0.87–0.89)	0.94(0.93–0.98)
High	69 558	23 236 (33.4)	0.71(0.70–0.72)	0.92(0.90–0.94)	1.03(1.00–1.05)
Information from hospital registers					
Admission to a psychiatric ward	2131	1356 (1.3)	2.53 (2.40–2.67)	1.51(1.35–1.68)	2.20 (2.06–2.36)

SD, standard deviation; BPP, Børge Prien's Prøve (the military intelligence test). * Model including BPPs score, education and psychiatric hospital contact and stratified on birth year; ** per s.d. change in BPP-score.

### Use of benzodiazepines and cognitive decline

Table 2 shows participants’ characteristics overall and according to the cumulative benzodiazepine exposure during the last 22 years before re-examination. The mean age of participants at the re-examination was 64.4 [s.d. 4.2] years and the mean re-test interval was 44.0 years [s.d. 4.3]. Overall, 35.2% (*n* = 1823) of the men had filled at least one prescription of benzodiazepines. Nearly one-third of these men (31.8%) had a cumulative use of benzodiazepine below 31 DMEs, whereas 43.7% had the highest level of exposure (>120 DME). Participants with the highest exposure had the highest proportion of psychiatric hospital contacts prior to the index date.

**Table 2.** Characteristics of study participants (*n* = 5183 men) in relation to cumulative use of benzodiazepines at re-examination

		Ever use of benzodiazepines		Cumulative benzodiazepine over 22 years		
Variables	All participants	No	yes	1-30 DME	31-120 DME	>120 DME
*n* (%)	5183	3360 (64.2)	1823 (25.8)	580 (31.8)*	446 (24.5)*	767 (43.7)*
BPP score (mean [s.d.])						
Conscription	46.2 [9.0]	47.2 [9.2]	45.5[10.0]	47.1 [10.1]	46.3 [9.7]	44.6 [10.1]
Follow-up	42.7 [9.2]	43.5 [8.9]	41.1 [9.7]	42.1 [9.7]	42.3 [9.4]	39.8 [10.5]
BPP change	−4.0 [6.1]	−3.7 [5.9]	−4.4 [6.6]	−4.0 [6.5]	−4.1 [6.7]	−4.9 [6.0]
Minimal important decline **>½** s.d. (n,(%))	2363 (45.6)	1477 (44.0)	886 (48.6)	266 (47.5%)	198 (49.5)	422 (57.8)
Age at examination, years (mean [s.d.])						
Conscription	20.4 [2.1]	20.4 [2.2]	20.3 [2.1]	20.5 [2.2]	20.2 [2.2]	20.4 [2.1]
Follow-up	64.4 [4.1]	64.3 [4.0]	64.5 [3.9]	64.8 [4.1]	64.6 [3.7]	64.3 [3.7]
Retest interval length	44.0 [4.3]	43.9 [4.4]	44.2 [4.1]	44.3 [4.3]	44.4 [4.2]	43.9 [3.9]
Year of birth (mean [s.d.])	1954.1 [3.2]	1954.3 [3.2]	1953.8 [3.0]	1953.7 [3.1]	1953.7 [2.9]	1953.4 [3.0]
Years of education (mean [s.d.])	13.7 [2.5]	13.8 [2.5]	13.6 [2.6]	13.7 [2.6]	13.6 [2.7]	13.4 [2.0]
Smoking						
Never (*n* (%))	1696 (32.3)	1185 (35.4)	511 (28.1)	173 (29.9)	121 (27.3)	511 (28.2)
Ever (*n* (%))	3470 (67.2)	2166 (64.6)	1312 (71.9)	405 (70.1)	322 (72.7)	577 (72.7)
Average alcohol consumption**						
0 units of alcohol per week (*n* (%))	271 (5.2)	163 (4.9)	108 (6.0)	32 (5.5)	23 (5.2)	53 (6.7)
<10 units of alcohol a week (*n* (%))	3178 (61.5)	2162 (64.3)	1016 (56.0)	334 (57.8)	252 (57.3)	430 (54.2)
>10 units of alcohol a week (*n* (%))	1718 (33.3)	1026 (30.9)	696 (38.1)	213 (35.8)	168 (36.8)	311 (39.2)
Psychiatric hospital contact***						
Yes (*n* (%))	329 (6.4)	130 (3.9)	199 (10.9)	41 (7.1)	29 (6.5)	129 (16.2)
No (*n* (%))	4854 (93.7)	3230 (96.1)	1624 (89.1)	539 (92.9)	417 (93.5)	668 (83.8)

* % of benzodiazepines users.

** Average alcohol consumption per week age 30–40 years.

*** Any psychiatric hospital contact before 1995; DME, Diazepam milligram equivalents.

**Figure 1.**
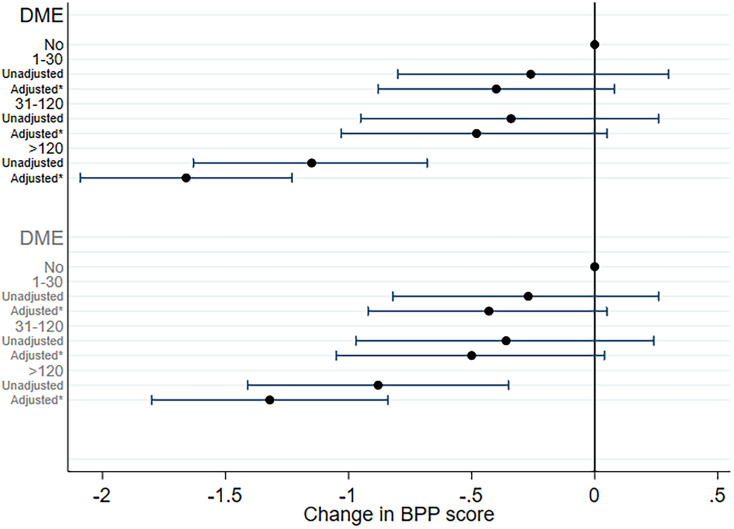
Association (beta-coefficients and 95% confidence intervals) between use of benzodiazepines in cumulative diazepam milligram equivalents (DME) (*n* = 5183) and cognitive decline measured with changes in Børge Priens Prøve (BPP) scores over 44 years. Legends in black concern the analysis with all users, whereas the gray legends are the analyses with current users excluded. *Adjusted for age at re-examination, re-test interval, BPP at conscription, years of education, alcohol use, smoking, and psychiatric hospital contact.

The average BPP score at conscription was 46.2 [s.d. 9.0] and declined to a mean of 42.7 [s.d. 9.2] at the re-examination, with slightly lower mean baseline scores and slightly larger decline across levels of cumulative benzodiazepine use (Table 2). In analysis with never users as reference, both the unadjusted and adjusted models showed a slightly larger decline among benzodiazepine users at the highest exposure level (>120 DME) compared to non-users (Fig. 1, top). Benzodiazepine use at or below 120 DME was not associated with cognitive decline. The percentage of variance (*R*2) in cognitive decline explained by benzodiazepine use was 0.01%. In the adjusted model, benzodiazepine use was associated with larger cognitive decline in the men with a cumulative use above 120 DME (*β*-coefficient [95% CI] = −1.66 [−2.09 to −1.23] BPP scores) compared with the decline of never users. Moreover, the 211 men who were defined as current users at the time of re-examination had larger cognitive decline than never users (*β*-coefficient [95% CI] = −2.42 [−3.18 to −1.66] BPP scores) and previous users (*β*-coefficient [95% CI] = −1.66 [−2.51 to −0.81] BPP scores). When current users were excluded from the analysis, men with cumulative use above 120 DME had a −1.32 *β*-coefficient (95% CI −1.80, −0.84) larger decline than the decline among those using 1–30 DME after adjustments (Fig. 1, bottom).

Among benzodiazepine users, equal proportions had used Z-drugs, benzodiazepines or both types of medications (Table 3). Users of both Z-drugs and benzodiazepines had a larger cognitive decline than never users (*β*-coefficient [95% CI] = −1.34 [−1.82 to −0.87] BPP scores). They also showed a larger decline than men who had only used Z-drugs (*β*-coefficient [95% CI] = −0.91 [−1.58 to −0.24] BPP scores) but this difference vanished when the cumulative dose was adjusted for (*β*-coefficient [95% CI] = −0.38 [−1.11 to 0.33]).

**Table 3.** Change in Børge Prien's Prøve (BPP) score (*n* = 5183 men) in relation to current and type of benzodiazepine use at re-examination

		Current use of benzodiazepines		Type of benzodiazepines used		
Variables	No users	No	Yes	Z-drugs only	Benzodiazepines, only	Both
*n* (%)	3360 (64.2)	1608 (88.8)	215 (11.2)	590 (32.9)	579 (32.3)	626(34.9)
BPP score (mean [s.d.])						
Conscription	47.2 [9.2]	45.7 [9.9]	44.3 [10.5]	47.0 [9.3]	45.1 [10.8]	44.5 [10.0]
Follow-up	43.5 [8.9]	41.4 [9.5]	38.8 [10.9]	42.8 [8.8]	40.6 [10.1]	40.1 [9.9]
BPP change	−3.7 [5.9]	−4.3 [6.6]	−5.5 [6.3]	−4.2 [6.5]	−4.5 [6.9]	−4.5 [6.5]
Minimal important decline **>½** s.d. (*n* (%))	1477 (44.0)	775 (48.2)	111 (51.6)	285 (48.3%)	280 (48.4)	306 (48.9)
Change* in BBP score compared to no users	0	−0.77 (−1.32-0.04)	−2.42 (−3.18 to −1.66)	−0.46(−0.94- 0.02)	−1.09(−1.58 to −0.60)	−1.34(−1.82 to −0.87)
Change* in BBP score within benzodiaepine users		0	−1.66(−2.51 to −0.81)	0	−0.63 (−1.32 to 0.05)	−0.91(−1.58 to −0.24)

* Beta-coefficients from multiple linear regression model with age at re-examination, re-test interval, BPP at conscription, years of education, alcohol use, smoking, psychiatric hospital contact.

However, all the above estimated differences were smaller than ¼ s.d. and thus did not reach the predefined cut-off point for a relevant difference of ½ s.d. = 4.5 BPP score points.

## Discussion

In this cohort of 335 513 men, we found that lower cognitive ability in young adulthood was associated with a higher risk of initiating benzodiazepines during adult life. When a subsample of 5183 men was re-examined on average 44 years later, those with the highest cumulative use of benzodiazepines (>120 DME) showed a larger decline in cognitive ability compared to men who had never used benzodiazepines or those with the lowest use. However, the estimated differences in cognitive decline did not reach the ½ s.d. cutoff point for a relevant difference.

We are the first to investigate and show a higher risk of purchasing benzodiazepines among men with lower cognitive ability in young adulthood. Our finding supports our first hypothesis and is in line with studies that have shown an inverse association between cognitive ability and risk of mental disorder (Christensen, Rozing, Mortensen, Christensen, & Osler, 2018; Gale et al., 2008; Osler et al., 2007b; Zammit et al., 2004) and might reflect that cognitive ability is related to symptoms that can be part of mental disorders (Osler et al., 2007b). Concerning the second hypothesis, our study is not comparable with previous longitudinal studies on benzodiazepine use and cognitive decline as we have more than 4 times longer re-test interval and a substantially longer exposure period. Thus, our results do not fully align with the most recent studies where a high cumulative dose of benzodiazepines was not associated with cognitive decline (Gray et al., 2016; Mura et al., 2013). A study from Seattle, US, where 3434 participants with a mean baseline age of 74 years were followed for benzodiazepine exposure over a 10-year period found no difference in mean cognitive score or rates of decline. The French Three-City Study followed 5195 participants with a mean age of 75 years at baseline for 7 years and showed that chronic benzodiazepine use was not associated with cognitive decline but with lower cognitive level at baseline and follow-up examinations in cross-sectional analysis. However, the study from Seattle did not indicate how large the overall decline in cognitive ability was between baseline and follow-up and in the French study the decline was below 1 point in the Mini-Mental State Examination test. In another French cohort (*n* = 1176 subjects aged 60–70 years at baseline), 63 subjects who reported benzodiazepine use in three consecutive examinations over the 4 years of follow-up presented an accelerated decline in cognitive performance compared to non-users (Paterniti, Dufouil, & Alpérovitch, 2002). Comparable with the findings in our study, lower levels of benzodiazepine use – defined as episodic or recurrent use – were not associated with cognitive decline. We also showed that current benzodiazepine users had a larger cognitive decline compared with non- or previous users. This result is also in contrast with that from the Seattle cohort which showed no cognitive difference between recent and non-recent users (Gray et al., 2016), whereas in the two French cohorts (Mura et al., 2013; Paterniti et al., 2002) chronic and thus recent benzodiazepine users had lower cognitive test scores compared to non-users. It has been proposed that the duration of action of benzodiazepines is directly correlated with cognitive decline (Picton et al., 2018). However, the evidence is scarce and derives from cross-sectional studies with single measurements of cognitive ability (Helmes & Østbye, 2015) or from the French three-city cohort where the outcome was Instrumental activity of daily living (IADL) limitation incidence (Carrière et al., 2015). Our study indicated that men with an exclusive use of short-acting Z-drugs had less cognitive decline than users with a combined use of both long and short-acting benzodiazepines. However, the association disappeared when the difference in dose was adjusted for several confounders, and we also cannot exclude that any found association will reflect the effect of the underlying conditions that benzodiazepines are prescribed for rather than the medication.

Finally, it should be noted that all the differences in change in cognitive scores we found were relatively small and did not reach the cutoff point for a relevant difference of ½s.d. = 4.5 BPP score points. However, the choice of the cutoff point for a minimal relevant difference is open to discussion. In cognitive research, there is a risk of not recognizing weak, yet important associations if cutoff points are set too high (Götz, Gosling, & Rentfrow, 2022). Conversely, using a sufficiently high cutoff point helps to account for the possibility that weak correlations might often be explained by unmeasured confounding and imperfect re-test reliability (Primbs et al., 2023; Revicki et al., 2008). In our study, we cannot exclude that the association between benzodiazepine use and cognitive decline may be influenced by the indication for which the medication was prescribed. The only indication for Z-drugs in Denmark is insomnia, while anxiety or sleep disorders are indications for benzodiazepines. Most prescriptions are likely due to insomnia or anxiety which are common symptoms of depression. Although we were able to adjust for depression, we had no baseline information on insomnia. Poor sleep has been associated with poorer cognitive performance during a dual-task test (Roth, 2007).

### Strengths and limitations

The present study was based on cognitive ability measured at the mandatory conscription board examination in young adulthood on a relatively large population-based sample that was followed into old age and of whom around 30% had at least one purchase of benzodiazepines registered in the Danish National Prescription Registry. Thus, benzodiazepine use as outcome (hypothesis 1) or exposure (hypothesis 2) were analysed by computerized pharmacy data, recorded independently of the participants' memory. This excluded selective reporting and the risk of bias due to unsystematic misclassification. Besides the availability of prospectively collected data from registries, the DanACo cohort also has other strengths compared to existing cognitive ageing cohorts such as a large sample size, a global measure of cognitive ability administered both at conscription and at the re-examination, and a long re-test interval (mean 44.0 years). Although we controlled for several potentially confounding factors, we admittedly could not preclude the possibility of unmeasured confounding associated with the use of benzodiazepines. Many of the data collected prior to the initiation of benzodiazepines were register-based and the information on psychiatric hospital contacts prior to the index date may not fully reflect the participants' mental health status, which may influence the initiation of benzodiazepine treatment and also cognitive decline (Zhao et al., 2022). Moreover, our measure of benzodiazepine use did not fully capture adult exposures as the Danish National Prescription Registry was started in 1995 when the mean age of our study sample was 35–45 years. This may have resulted in some misclassification of our exposure/outcome measures and might have led to an underestimation of the risk estimates, assuming that the misclassification was random. Another weakness of the DanACo cohort is the low participation rate (14.3%) but our study showed that despite the high non-response rate and differences between non-responders and responders on baseline characteristics, there were no overt effects of non-response on risk estimates for associations of young adult cognitive ability and benzodiazepine use. Our previous studies have shown that men with low education, low cognitive ability, and low or high BMI at conscription have a higher risk of premature mortality (Christensen, Mortensen, Christensen, & Osler, 2016; Jorgensen et al., 2016). Thus, as all studies based on re-examinations in middle age, these groups may be underrepresented in our population. Finally, our cohort consisted of men only, hindering generalization to women. However, previous studies linking benzodiazepines with cognitive decline did not suggest sex differences in risk estimates.

In conclusion, low early-life cognitive ability increases the risk of benzodiazepine use later in life, and mean cognitive decline seems to be larger in the highest level of benzodiazepine use compared with non-use, but the difference was small and did not reach the cut-off point for a clinically relevant difference. This indicates that already well-documented and important risk factors for cognitive impairment and dementia such as low education, alcohol abuse, and depression (Livingston et al., 2017; Walhovd, Lövden, & Fjell, 2023), which are closely associated with benzodiazepines, may be more relevant for preventive efforts.

## Supporting information

Osler et al. supplementary material Osler et al. supplementary material

## Data Availability

The data that support the findings of this study are available from Statistics Denmark. Restrictions apply to the availability of these data, which were used under license for this study.

## Supplementary material

The supplementary material for this article can be found at https://doi.org/10.1017/S0033291724002046.
