# Supplementary material for: Associations of benzodiazepine use with cognitive ability and age-related cognitive decline
Source: Psychol Med. 2024 Oct 14;54(13):3729–36. doi: 10.1017/S0033291724002046 (PMC11536101; doi:10.1017/S0033291724002046)
Supplement: Osler et al. supplementary material [file S0033291724002046sup001.docx]

**Supplementary Table 1. Associations of cognitive ability in** **young adulthood and use of benzodiazepines in middle age among participants and non-participants in DanACo**

|  | Non-Participants | | Participants |  |
| --- | --- | --- | --- | --- |
|  | Benzodiazepine  users  n (% of total) | Adjusted* hazard ratio  (95% CI) | Benzodiazepine  users  n (% of total) | Adjusted* hazard ratio  (95% CI) |
| Total | 13 266 (43.2)** | - | 1863 (36.2)** | - |
| Birth year, Mean [SD] | 1954 [3.0] | - | 1954 [3.0] | - |
| **Conscription board examination** |  |  |  |  |
| *BPP-score*, mean [SD] | 38.5 [11.5] | 0.85(0.83-0.87)** | 46.6 [9.1] | 0.85(0.80-0.91)** |
| Low | 5,033 (37.9) | Ref | 305 (16.3) | Ref |
| Medium | 4,276 (32.2) | 0.86 (0.82-0.91) | 539 (28.9) | 0.77(0.66-0.87) |
| Hight | 3,952 (29.8) | 0.72 (0.63-0.76) | 1020 (54.7) | 0.76 (0.65-0.87) |
| *Educational level* |  |  |  |  |
| Low | 3158 (23.8) | Ref | 165 (8.9) | Ref |
| Medium | 6913 (52.2) | 0.91(0.86-0.95) | 916 (49.2) | 0.84(0.70-1.00) |
| High | 3173 (24.0) | 1.02 (0.95-1.08) | 779 (41.9) | 0.88(0.71-1.06**) |
| **Information from hospital registers** |  |  |  |  |
| Admission to a psychiatric ward | 317 (2.4) | 1.47 (1.31-1.64) | 23 (1.2) | 1.34(0.88-2.05) |
|  |  |  |  |  |

Abbreviations: SD: Standard deviation; BPP = Børge Prien’s Prøve (the military intelligence test). * Estimates are mutually adjusted and stratified on birth year, **n and % of all Non-participants or Participants.** per SD increase in BPP

**Supplementary Table 2. Diazepam equivalent doses (DME)**

| **ATC code** |  | **DME** |
| --- | --- | --- |
| N05BA12 | Alprazolam | 10 |
| N05BA08 | Bromazepam | 1 |
| N05BA02 | Chlordiazepoxid | 0.5 |
| N05BA09 | Clobazam | 0.5 |
| N03AE01 | Clonazepam | 20 |
| N05BA01 | Diazepam | 1 |
| N05DC05 | Estazolam | 5.0 |
| N05BA06 | Lorazepam | 5.0 |
| N05CD06 | Lormetazepam | 5.0 |
| N05BA04 | Oxazepam | 0.5 |
| N05CD02 | Nitrazepam | 1 |
| N05CD05 | Triazolam | 40 |
| N05CF02 | Zolpidem | 0.5 |
| N05CF01 | Zopiclon | 1.33 |
| N05CF03 | Zaleplon | 0.5 |

**Example calculation:**

A standard prescription of Zolpidem would be 1 tablet (10 mg) daily. Resulting in an equipotent dose of 5 mg DME/daily with a conversion factor of 0.5.


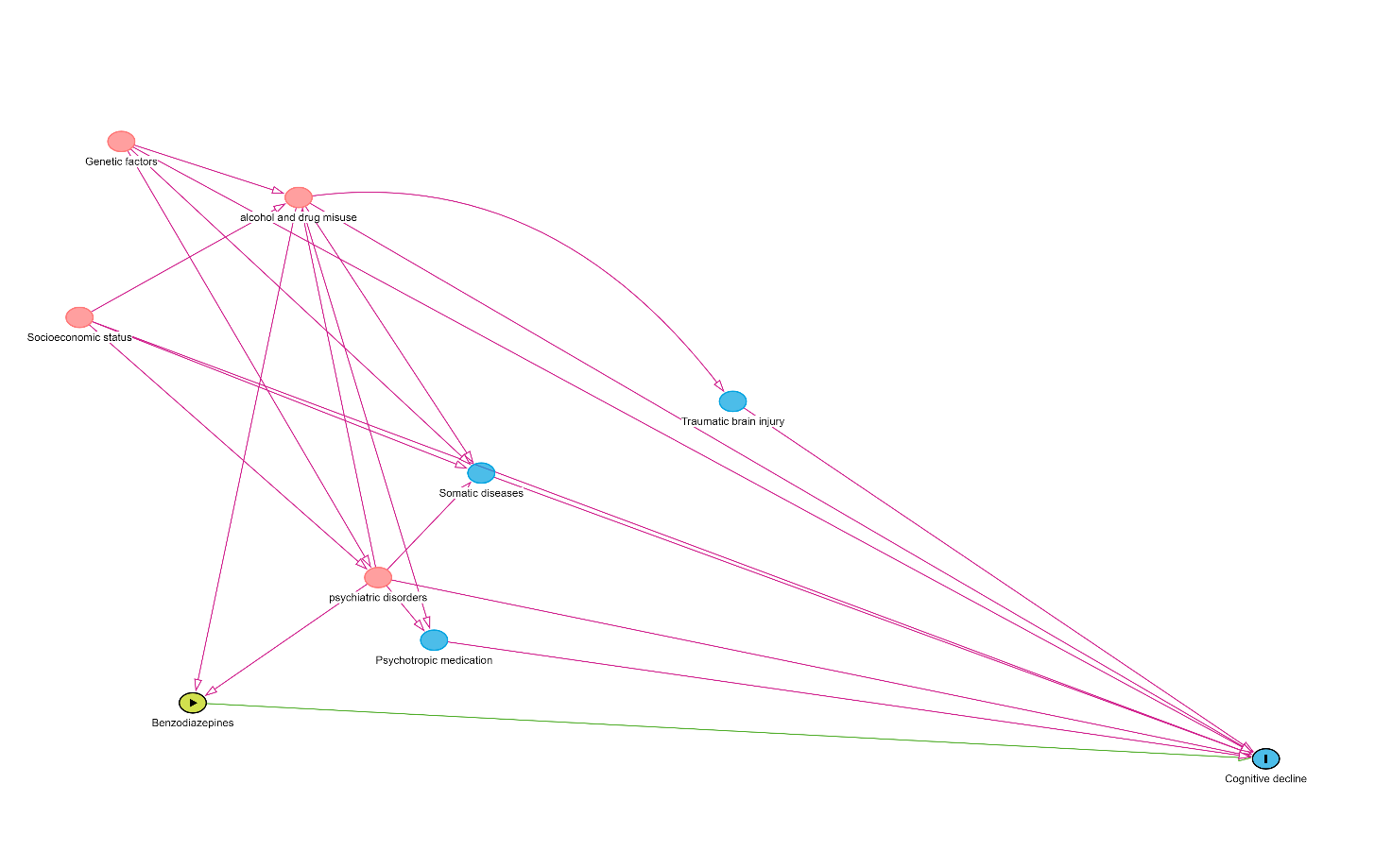


Supplementary Figure 1. Directed Acyclic Graph (DAG) for the association between benzodiazepines use and cognitive decline.
